# Rapid detection of *Salmonella* in food and feed by coupling loop-mediated isothermal amplification with bioluminescent assay in real-time

**DOI:** 10.1186/s12866-016-0730-7

**Published:** 2016-06-17

**Authors:** Qianru Yang, Kelly J Domesle, Fei Wang, Beilei Ge

**Affiliations:** Division of Animal and Food Microbiology, Office of Research, Center for Veterinary Medicine, U.S. Food and Drug Administration, Laurel, MD 20708 USA; Department of Food Science, Louisiana State University Agricultural Center, Baton Rouge, LA 70803 USA; Department of Nutrition and Food Science, University of Maryland, College Park, MD 20742 USA

**Keywords:** LAMP, BART, *Salmonella*, Food, Feed, Detection

## Abstract

**Background:**

*Salmonella* is among the most significant pathogens causing food and feed safety concerns. This study examined the rapid detection of *Salmonella* in various types of food and feed samples by coupling loop-mediated isothermal amplification (LAMP) with a novel reporter, bioluminescent assay in real-time (BART). Performance of the LAMP-BART assay was compared to a conventional LAMP and the commercially available 3M Molecular Detection Assay (MDA) *Salmonella*.

**Results:**

The LAMP-BART assay was 100 % specific among 178 strains (151 *Salmonella* and 27 non-*Salmonella*) tested. The detection limits were 36 cells per reaction in pure culture and 10^4^ to 10^6^ CFU per 25 g in spiked food and feed samples without enrichment, which were comparable to those of the conventional LAMP and 3M MDA *Salmonella* but 5–10 min faster. Ground turkey showed a strong inhibition on 3M MDA *Salmonella*, requiring at least 10^8^ CFU per 25 g for detection. The correlation between *Salmonella* cell numbers and LAMP-BART signals was high (*R*^2^ = 0.941–0.962), suggesting good quantification capability. After 24 h enrichment, all three assays accurately detected 1 to 3 CFU per 25 g of *Salmonella* among five types of food (cantaloupe, ground beef, ground turkey, shell eggs, and tomato) and three types of feed (cattle feed, chicken feed, and dry dog food) examined. However, 10^1^ CFU per 25 g was required for cattle feed when tested by 3M MDA *Salmonella*.

**Conclusions:**

The *Salmonella* LAMP-BART assay was rapid, specific, sensitive, quantitative, and robust. Upon further validation, it may become a valuable tool for routine screening of *Salmonella* in various types of food and feed samples.

## Background

Nontyphoidal *Salmonella* is a zoonotic agent of significant food and feed safety concerns. In the United States, an estimated 1 million cases of foodborne salmonellosis occur each year, resulting in the highest numbers of hospitalizations and deaths among 31 major pathogens [1]. *Salmonella* also represented the leading cause of foodborne disease outbreaks during 1998–2012, with 77 % of illnesses broadly attributed across multiple food commodities, including produce, eggs, poultry, and meats [2]. Moreover, *Salmonella* remains a major microbial hazard in animal feed and pet food [3]. The safety of these feed commodities impacts not only animal health but also the health of humans consuming foods of animal origin or handling pet food [4]. For instance, several multistate outbreaks of human salmonellosis linked to tainted pet food have been reported recently [5].

To reduce *Salmonella* outbreaks and illnesses associated with food and feed products, a multifaceted approach from farm to table is required. Methods that can quickly and reliably detect *Salmonella* in these commodities are especially valuable in order to promptly identify contamination problems along the production chain. However, rapid, reliable, and robust detection of *Salmonella* in food and feed remains elusive [6]. Conventional culture methods are reliable but time consuming and labor intensive, taking days even weeks for a definitive result [7]. A growing number of molecular methods, including PCR and real-time quantitative PCR (qPCR) have been developed and applied to detect *Salmonella* in a variety of food and feed products [8–11]. Despite being rapid, specific, and sensitive, PCR-based nucleic acid amplification tests (NAATs) require a sophisticated thermal cycling instrument and are also susceptible to inhibitors in food and feed matrices [12, 13], limiting their wider application.

Recently, an isothermal NAAT termed loop-mediated isothermal amplification (LAMP) has emerged as a promising alternative to PCR for the detection of *Salmonella* in food [14–17]. LAMP employs four to six specially designed primers and a strand-displacing *Bst* DNA polymerase to amplify up to 10^9^ copies of target DNA within an hour [18, 19]. Two distinct advantages of LAMP over PCR are running at a constant temperature (~65 °C) and tolerance to assay inhibitors [20, 21], which eliminate the need for a thermocycler or complicated sample preparation steps. Other attractive features of LAMP include high specificity, sensitivity, speed, and robustness [16, 22]. Nonetheless, LAMP has not yet been evaluated in feed samples, which encompass a group of rather diverse and complex matrices.

Efficient sample analysis with LAMP depends not only on the performance of DNA amplification but also the method used for monitoring the reaction [23]. To date, multiple techniques have been used to detect LAMP products, including naked eye, gel electrophoresis, turbidity, fluorescence, among others [23]. Bioluminescent monitoring of LAMP products was demonstrated recently via a novel reporter, bioluminescent assay in real-time (BART) [24]. In essence, BART monitors the inorganic pyrophosphate produced during the LAMP reaction by converting it to ATP which is simultaneously utilized by firefly luciferase to emit light [24]. The time needed to reach peak light output is reflective of the concentration of original target DNA; therefore, LAMP-BART allows real-time quantification with a simple, portable light detector [24]. When applied in detecting genetically modified maize, LAMP-BART was shown to be an effective and sensitive technique with significant potential for quantification [25]. A commercially available 3M molecular detection assay (MDA) *Salmonella* (3M Food Safety, St. Paul, MN) also builds upon the LAMP-BART technology.

In this study, we aimed to develop and optimize an in-house *Salmonella* LAMP-BART assay and to apply the assay in various types of food and feed samples. The assay’s performance was compared with that of a conventional LAMP assay and the commercially available 3M MDA *Salmonella*.

## Results

### The optimized LAMP-BART assay

**Fig. 1:**
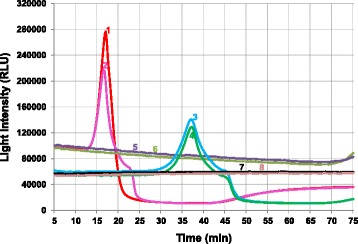
LAMP-BART amplification graphs generated when running the assays with optimized or prototypic reagent mixes. Samples 1 to 2 and 3 to 4 were run using optimized and prototypic [24, 25] reagent mixes, respectively. Sample 5 to 6 and 7 to 8 were water samples run using the optimized and prototypic mixes, respectively

The final LAMP-BART reaction mix in a total volume of 25 μl contained all core reagents listed in the Methods section and two (polyvinylpyrrolidone (PVP) and trehalose) out of the four facilitators evaluated. KCl was excluded from the mixture due to significantly greater *T*_*max*_ values when added individually or in combination with other facilitators (*P* < 0.001). Dithiothreitol (DTT) was not included since it did not improve the overall assay performance in terms of *T*_*max*_ values or false positive rates. It is noteworthy that trehalose, when added alone or together with other facilitators, consistently gave the optimum assay performance. Fig. 1 shows the amplification graphs when running the assays with the optimized reagent mix compared to the prototypic one. Besides decreased *T*_*max*_ values (17 versus 37 min), the light intensity was also greater using the optimized mix.

### Assay specificity

All three assays (LAMP-BART, conventional LAMP, and 3M MDA *Salmonella*) demonstrated 100 % specificity when testing the 178 bacterial strains. For the 151 *Salmonella* strains of 100 serovars, the mean *T*_*max*_ values ranged from 8.1 to 17 min with an average of 11.1 ± 1.6 min by LAMP-BART and from 14.8 to 27 min with an average of 18 ± 2.3 min by 3M MDA *Salmonella*, whereas the mean *T*_*t*_ values ranged from 12.6 to 25.4 min with an average of 15 ± 2.3 min by conventional LAMP. The overall ranking of assay rapidity was LAMP-BART > conventional LAMP > 3M MDA *Salmonella* (*P* < 0.0001). For the 27 non-*Salmonella* strains, no *T*_*max*_ or *T*_*t*_ value was obtained, suggesting negative results by all three assays.

### Assay sensitivity and quantification capability

**Fig. 2:**
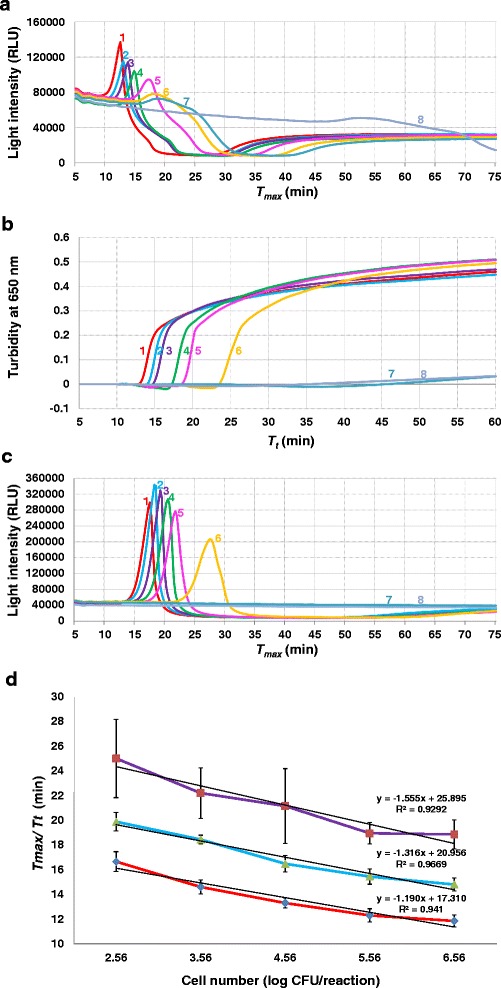
The sensitivity and quantification capability of LAMP-BART, conventional LAMP, and 3M MDA *Salmonella* when testing 10-fold serial dilutions of *Salmonella enterica* serovar Typhimurium LT2 cultures. (**a**-**c**) Representative amplification graphs obtained by LAMP-BART (**a**), conventional LAMP (**b**), and 3M MDA *Salmonella* (**c**). Samples 1 to 7 correspond to 10-fold serial dilutions of *S*. Typhimurium LT2 cells ranging from 3.6 × 10^6^ to 3.6 CFU/reaction; sample 8 is water. (**d**) Standard curves generated based on five independent repeats by LAMP-BART (*bottom*), conventional LAMP (*middle*), and 3M MDA *Salmonella* (*top*)

Figure 2 presents the sensitivity and quantification capability of these assays when testing 10-fold serial dilutions of *S*. Typhimurium LT2 DNA templates ranging from 3.6 × 10^6^ to 3.6 CFU/reaction. Representative amplification graphs and corresponding standard curves are shown in Fig. 2a-c and d, respectively. All three assays consistently detected down to 36 CFU of *Salmonella* per reaction in five repeats, with average *T*_*max*_ values ranging from 11.9 to 18.1 min by LAMP-BART and from 18.9 to 37.9 by 3M MDA *Salmonella*, and average *T*_*t*_ values ranging from 14.8 to 25.2 min by conventional LAMP. In three out of five repeats, LAMP-BART and conventional LAMP also detected 3.6 *Salmonella* cells per reaction while 3M MDA *Salmonella* detected this level in two repeats (data not shown).

Based on the standard curves generated (Fig. 2d), linear relationships were observed for templates ranging from 3.6 × 10^6^ to 3.6 × 10^2^ CFU/reaction with correlation coefficients (*R*^*2*^) at 0.941, 0.967, and 0.929 for LAMP-BART, conventional LAMP, and 3M MDA *Salmonella*, respectively. When taking into consideration the 36 CFU/reaction level, the *R*^*2*^ values dropped to 0.875 and 0.727 for conventional LAMP and 3M MDA *Salmonella*, respectively, but increased to 0.962 for LAMP-BART (data not shown).

### Rapid and sensitive detection of *Salmonella* in spiked food and feed samples

All of the uninoculated controls tested negative for *Salmonella* (data not shown). Aerobic plate counts among food types averaged 10^2^-10^3^ CFU/g in cantaloupe and tomato, 10^4^ CFU/g in ground beef and ground turkey, and non-detectable (< 10^2^ CFU/g) in shell eggs. Among feed types, the aerobic plate counts averaged 10^2^ CFU/g in dog food, 10^3^ CFU/g in chicken feed, and 10^4^ CFU/g in cattle feed.

Table 1 summarizes the sensitivity of all three assays when testing 10-fold serial dilutions of *Salmonella* strains of various serovars in spiked food and feed samples based on three independent repeats. In the majority of food types, the detection limits for LAMP-BART and conventional LAMP were around 2 × 10^4^ CFU per 25 g (*ca*. 8 × 10^2^ CFU/g, equivalent to 1.6 CFU/reaction) except in ground turkey and ground beef, for which the detection limits were 10-fold higher. In one or two out of three repeats, conventional LAMP and LAMP-BART, respectively, achieved positive results in ground beef at the 2× 10^4^ CFU/25 g level. The 3M MDA *Salmonella* assay was capable of detecting 10^4^ CFU per 25 g in shell eggs and tomato in some repeats but required at least 10^5^ CFU/25 g in cantaloupe and ground beef and as high as 10^8^ CFU/25 g in ground turkey. In feed samples, regardless of feed type, all three assays required at least 10^5^ CFU/25 g for detection and 10^6^ CFU/25 g was consistently needed to detect *Salmonella* Newport 1240 H in cattle feed by 3M MDA *Salmonella* (Table 1).

**Table 1:** The sensitivity of LAMP-BART, conventional LAMP, and 3M MDA *Salmonella* in spiked food and feed samples based on three independent repeats

Food or feed type	*Salmonella* serovar	Detection limit (CFU/25 g) ^a-b^ without enrichment		
		LAMP-BART	Conventional LAMP	3M MDA *Salmonella*
Food samples				
Cantaloupe	Poona	2.0 × 10^4^	2.0 × 10^4^	2.0 × 10^5^
Ground beef	Typhimurium	2.0 × 10^5b^	2.0 × 10^5a^	2.0 × 10^6a^
Ground turkey	Heidelberg	1.7 × 10^5^	1.7 × 10^5^	1.7 × 10^8^
Shell eggs	Enteritidis	1.7 × 10^4^	1.7 × 10^4^	1.7 × 10^5b^
Tomato	Typhimurium	2.0 × 10^4^	2.0 × 10^4^	2.0 × 10^5a^
Feed samples				
Cattle feed	Newport	1.7 × 10^5^	1.7 × 10^5^	1.7 × 10^6^
Chicken feed	Enteritidis	1.7 × 10^6b^	1.7 × 10^6b^	1.7 × 10^6a^
Dry dog food	Infantis	1.1 × 10^5^	1.1 × 10^5^	1.1 × 10^6a^

^a-b^In one (a) or two (b) out of three repeats, the assays detected respective *Salmonella* serovars at concentrations 10-folder lower than those presented

**Fig. 3:**
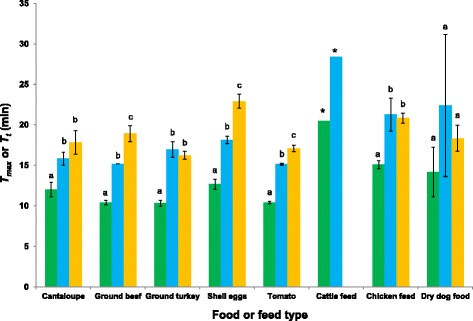
Comparison of LAMP-BART (*green bars*), conventional LAMP (*blue bars*), and 3M MDA *Salmonella* (*yellow bars*) in detecting low-level (1 to 3 CFU/25 g) *Salmonella* strains of various serovars in spiked food and feed samples after 24 h enrichment based on three independent repeats. * In cattle feed, positive detection of *Salmonella* Newport 1240 H only occurred in one repeat each by LAMP-BART and conventional LAMP but none by 3M MDA *Salmonella*. Bars labeled with different *lower case letters* within the same food or feed type indicate statistically significant *T*_*max*_ or *T*_*t*_ values generated by LAMP-BART, conventional LAMP, and 3M MDA *Salmonella* (*P* < 0.05)

Figure 3 shows the detection of low-level (1 to 3 CFU/25 g) *Salmonella* of various serovars in spiked food and feed samples after 24 h enrichment based on three independent repeats. Regardless of food or feed type, LAMP-BART consistently gave the lowest *T*_*max*_ values compared to the other two assays (*P* < 0.0001). For the vast majority of food and feed types, all three assays achieved successful detection in all three repeats. In cattle feed, positive detection of *Salmonella* Newport 1240 H only occurred in one repeat each by LAMP-BART and conventional LAMP but none by 3M MDA *Salmonella*. When 10-fold higher concentration of this strain was spiked in cattle feed, positive results were returned in all three repeats with mean *T*_*max*_ values of 17.8 and 27.1 min for LAMP-BART and 3M MDA *Salmonella*, respectively, and a mean *T*_*t*_ value of 22.2 min for conventional LAMP (data not shown). Interestingly, *T*_*max*_ or *T*_*t*_ values obtained by all three assays were significantly higher when testing feed types compared to food types (*P* < 0.001).

## Discussion

Coupling a promising isothermal NAAT with a novel bioluminescent reporter, the *Salmonella* LAMP-BART assay developed in this study was rapid (8–45 min), specific (100 % inclusivity and exclusivity among 178 strains tested), sensitive (36 cells/reaction in pure culture and 10^4^-10^6^ CFU/25 g in spiked food and feed), quantitative (*R*^2^ = 0.941–0.962), and robust (applicable in eight types of food or feed matrices). After 24 h enrichment, the assay accurately detected 1–3 CFU/25 g of *Salmonella* in all food/feed types tested except cattle feed. This is the first study evaluating LAMP-BART for *Salmonella* detection in a large variety of food and feed samples.

Previously, the LAMP-BART combination has been successfully explored in detecting *Chlamydia trachomatis*, *Clostridium difficile*, and GMO [24–26] and forms the basis of several commercially available 3M MDAs [27]. A major advantage of BART over fluorescence or turbidity monitoring of LAMP reactions is the requirement of rather simple, robust, and low-cost hardware. For instance, a portable photodiode-based reader (Lumora Ltd, Cambridgeshire, UK) was used in the *C. trachomatis*, *C. difficile*, and GMO studies [24–26]. In the present study, the assay was conducted in a one-step, closed-tube format in the 3M MDS100 instrument providing temperature control (60 °C) for LAMP and bioluminescence readings for BART simultaneously. This feature of BART may potentially lead to the development of field-deployable assays that can be used in resource-limited areas.

The LAMP portion of the assay was essentially the same as the conventional LAMP included for comparison, which used slightly modified primer sequences (Table 2) from those published previously [14]. These modifications were necessary based on preliminary evaluation of the combined LAMP-BART performance (data not shown). It is notable that multiple LAMP assays have been recently developed and applied in detecting *Salmonella* in food, and reported to be rapid, specific, and sensitive [14–17, 28–30]. The *invA*-based LAMP was chosen in this study as the prototype for assay development and comparison purposes since it has been extensively evaluated in eggs and produce, and for robustness and comparison with qPCR [20, 31, 32].

**Table 2:** Primers used for detecting *Salmonella* by LAMP-BART and conventional LAMP, in comparison with the primer set published previously

Primer name	Sequence (5′-3′) ^a^	Position ^b^	Reference
Primer set optimized for LAMP-BART and also used in conventional LAMP			This study
Sal4-F3	GAACGTGTCGCGGAAGTC	484-501	
Sal4-B3	CGGCAATAGCGTCACCTT	665-682	
Sal4-FIP	GCGCGGCATCCGCATCAATA- TCTGGATGGTATGCCCGG	573-592 (F1c) 516-533 (F2)	
Sal4-BIP	GCGAACGGCGAAGCGTACTG- TCGCACCGTCAAAGGAAC	593-612 (B1c) 635-652 (B2)	
Sal4-Loop-F	TCAAATCGGCATCAATACTCATCTG	538-562	
Sal4-Loop-B	AAAGGGAAAGCCAGCTTTACG	614-634	
Primer set published in a previous study			[14]
F3	CGGCCCGATTTTCTCTGG	503-520	
B3	CGGCAATAGCGTCACCTT	665-682	
FIP	GCGCGGCATCCGCATCAATA- TGCCCGGTAAACAGATGAGT	573-592 (F1c) 527-546 (F2)	
BIP	GCGAACGGCGAAGCGTACTG- TCGCACCGTCAAAGGAAC	593-612 (B1c) 635-652 (B2)	
Loop-F	GGCCTTCAAATCGGCATCAAT	547-567	
Loop-B	GAAAGGGAAAGCCAGCTTTACG	613-634	

^a^Underlined sequences were either F2 or B2 as indicated. ^b^The positions are numbered based on the coding sequence of the *Salmonella invA* gene [GenBank: M90846]

Besides LAMP reagents, the optimized LAMP-BART reaction mix contained four essential reagents for BART (adenosine 5′ phosphosulfate (APS) and ATP sulfurylase to convert inorganic pyrophosphate produced during the LAMP reaction to ATP, and luciferin and firefly luciferase to utilize ATP to generate light) and two (PVP and trehalose) out of four facilitators (DTT, KCl, PVP, and trehalose) described previously [24, 25]. This is the first study evaluating the effects of these facilitators on LAMP-based assays. In PCR reactions, both DTT and trehalose enhanced amplification efficiency by stabilizing the *Taq* DNA polymerase, while trehalose also lowered DNA melting temperature [33, 34]. PVP has been shown to enhance PCR by reversing the inhibitory effect of polyphenolic contaminants [35]. Increasing KCl concentrations in the PCR buffer has been reported to cause preferential amplification of shorter DNAs as longer DNAs denatured slower due to the stabilizing effect of potassium ions on the double-stranded structure [36]. Our data suggested the inclusion of PVP and trehalose but not DTT or KCl. Notably, the intermediate and final LAMP products are a mixture of stem-loop DNAs with various stem lengths [18]. The amplification of longer ones was likely inhibited by increased KCl in the mix, as indicated by significantly greater *T*_*max*_ values when KCl was added.

In pure-culture testing, all three assays (LAMP-BART, conventional LAMP, and 3M MDA *Salmonella*) possessed similar specificity and sensitivity. LAMP-BART was consistently faster than conventional LAMP, while 3M MDA *Salmonella* was the slowest. Besides *T*_*max*_ (time to the maximum value of the light output curve), the time to the first inflexion point of the curve (*T*_*infl*_) could also be used to characterize the LAMP-BART amplification kinetics [24], further shortening the time taken to report positive results. The finding of 100 % specificity among 178 bacterial strains including all six subspecies of *S. enterica* and *S. bongori* corroborated previous reports on multiple *Salmonella* LAMP assays using various collections of bacterial strains [14, 15, 17, 28–32]. The detection limit of 36 CFU/reaction also fell within the range (1 to 40 cells per test) reported previously for multiple *Salmonella* LAMP assays [14, 15, 17, 20, 28–31]. The three LAMP-BART assays described recently had detection limits of 5.5 copies of *C. trachomatis* DNA, 10 copies of *C. difficile* DNA, and 40 copies of GMO target, respectively [24–26]. However, the detection limit of 3M MDA *Salmonella* in pure culture has not been reported.

Different from the sigmoidal shape typical of fluorescence and turbidity measurements, LAMP-BART possessed unique assay kinetics as shown in the bell-shape light output curves (Fig. 2), possibly leading to better quantification capability [24]. The *Chlamydia* LAMP-BART assay had an *R*^*2*^ of 1 for DNA templates ranging from 10^2^ to 10^8^ copies per reaction, but the linearity was greatly compromised for templates below 10^2^ copies [24]. In the present study, the LAMP-BART assay had *R*^*2*^ of 0.941 for *Salmonella* cells ranging from 10^2^ to 10^6^ CFU/reaction and the value increased to 0.962 when the 10^1^ CFU level was added. This was superior to either conventional LAMP or 3M MDA *Salmonella*. Previously, *R*^*2*^ for conventional LAMP was 0.97 for *Salmonella* cells ranging from 10^2^ to 10^5^ CFU/reaction [14]. The 3M MDA *Salmonella* was designed to be a qualitative assay, i.e., presence or absence; therefore, its quantification capability has not been examined previously.

Among most food/feed types tested, the three assays detected down to 2 × 10^4^ - 2 × 10^6^ CFU/25 g (equivalent to 1.6 to 160 CFU/reaction) without enrichment, while 10^8^ CFU/25 g was required by 3M MDA *Salmonella* for detection in ground turkey (Table 1). To our knowledge, this is the first time LAMP-based assays were evaluated in various feed samples and the second time in ground beef and ground turkey [37]. The reduced sensitivity (up to 100-fold) observed in these food/feed types suggested that relatively high background flora (indicated by APC) and/or complex matrices may have affected the assay performance. In particular, ground turkey with high average APC of 10^4^ CFU/g showed a strong inhibition on the 3M MDA *Salmonella* where all matrix control samples returned negative results. A recent study comparing 3M MDA *Salmonella* and ISO 6579 for the detection of *Salmonella* in retail meat samples also reported inhibition of the 3M assay by a turkey meat preparation (turkey meatball) with many ingredients, possibly due to spice [37]. The ground turkey used in the present study contained 7 % fat, which may have negatively influenced the 3M MDA *Salmonella* outcome.

Coupled with enrichment, the three assays accurately detected 1–3 CFU/25 g of *Salmonella* in all food/feed types tested except in cattle feed when tested by 3M MDA *Salmonella*, for which 10^1^ CFU per 25 g was required (Fig. 3). The findings in produce and shell eggs agreed with several recent reports on the capability of conventional LAMP in detecting low-level *Salmonella* in these food types [31, 32]. However, the detection of low-level *Salmonella* in feed samples has not been reported previously. Similar to the trend shown in food/feed sensitivity testing, all three assays were less effective (longer *T*_*max*_ or *T*_*t*_) in detecting feed samples than food samples, suggesting matrix effects caused by many ingredients commonly present in feed rations. This effect was even more apparent in cattle feed where only one repeat at the 10^0^ CFU/25 g level was positive by LAMP-BART and conventional LAMP and none by 3M MDA *Salmonella*. Another recent study using 3M MDA *Salmonella* in water sources also showed it to be less effective than PCR in detecting *Salmonella* [38]. It is hypothesized that natural flora present in cattle feed or compounds released during processing may have affected *Salmonella* survival and growth during enrichment, causing the low sensitivity in detection. Further studies are warranted to optimize detection in feed commodities. Finally, agreeable with pure-culture testing data, LAMP-BART was consistently faster than conventional LAMP, while 3M MDA *Salmonella* was the slowest when food/feed samples were tested.

## Conclusions

The *Salmonella* LAMP-BART assay developed in this study was rapid, specific, sensitive, quantitative, and robust. Upon further validation including independent validation and collaborative studies, it may become a valuable tool for routine screening of *Salmonella* in various types of food and feed samples.

## Methods

### Bacterial strains and culture conditions

*Salmonella* strains (*n* = 151) used in this study included all six subspecies (I, II, IIIa, IIIb, IV, and VI) of *Salmonella enterica* and *Salmonella bongori*, representing a total of 100 serovars. Non-*Salmonella* strains (*n* = 27) belonged to *Campylobacter*, *Citrobacter*, *Enterobacter*, *Escherichia coli*, *Hafnia*, *Listeria*, *Shigella*, and *Vibrio*. Detailed strain information was described previously [32]. Among *Salmonella* strains, *S. enterica* serovar Typhimurium LT2 was used for assay development and sensitivity testing, whereas *S. enterica* serovars Enteritidis S50, Heidelberg 1364 H, Infantis 1102 H, Newport 1240 H, Poona 2861 H, and Typhimurium LT2 were used in food and feed spiking experiments (Table 3). All bacterial strains were cultured on Trypticase soy agar or blood agar (BD Diagnostic Systems, Sparks, MD) at 35 °C overnight. *Campylobacter* strains were grown under microaerophilic conditions (85 % N_2_, 10 % CO_2_, and 5 % O_2_).

**Table 3:** *Salmonella* strains used in food and feed spiking experiments

*Salmonella* serovar	Strain	Food or feed inoculated	Origin	Source/reference ^a^
Enteritidis	S50	Shell eggs and chicken feed	Raw chicken	[41]
Heidelberg	1364 H	Ground turkey	Raw oysters	FDA, CFSAN
Infantis	1102 H	Dry dog food	Meat meal	FDA, CFSAN
Newport	1240 H	Cattle feed	Dried yeast	FDA, CFSAN
Poona	2861 H	Cantaloupe	Pet turtles	FDA, CFSAN
Typhimurium	LT2	Ground beef and tomato	Chicken	BEI Resources

^a^BEI Resources, National Institute of Allergy and Infectious Diseases/Biodefense and Emerging Infections Research Resources Repository; FDA, CFSAN, U.S. Food and Drug Administration, Center for Food Safety and Applied Nutrition

### LAMP-BART assay design and optimization

LAMP primers targeting the *Salmonella* invasion gene (*invA*; GenBank: M90846) were designed by using PrimerExplorer V4 (Fujitsu Limited, Japan). Each primer set consisted of two outer (F3 and B3), two inner (FIP and BIP), and one to two loop primers (Loop-F and/or Loop-B). The final primer set (Sal4) chosen for the LAMP-BART assay is shown in Table 2.

Based on the prototypic LAMP-BART reaction described previously [24, 25], the optimum components of the *Salmonella* LAMP-BART assay were evaluated by testing core reagents first followed by adding individual or a combination of four facilitators. The core reagent mix in a total volume of 25 μl contained 1× ThermoPol reaction buffer (New England Biolabs, Ipswich, MA), 6 mM MgSO_4_, 1.2 mM each deoxynucleoside triphosphate (dNTP), 0.1 μM F3 and B3 (Integrated DNA Technologies, Coralville, IA), 1.8 μM FIP and BIP, 1 μM Loop-F and Loop-B, 100 μg/ml luciferin potassium salt (Sigma-Aldrich, St. Louis, MO), 0.25 mM APS (Sigma-Aldrich), 0.5 U/ml ATP sulfurylase (New England Biolabs), 5.6 μg/ml Ultra-Glo firefly luciferase (Promega, Madison, WI), 10 U of *Bst* DNA polymerase (New England Biolabs), and 2 μl of DNA template (*S*. Typhimurium LT2 at 1.8 × 10^6^ CFU/ml). The four facilitators were DTT ( 10 mM), KCl (60 mM), PVP (0.4 mg/ml), and trehalose (87 mM), all obtained from Sigma-Aldrich. The optimization experiments were run in duplicate and repeated three times.

The LAMP-BART reaction was carried out at 60 °C for 75 min in the 3M Molecular Detection System instrument MDS100 (3M Food Safety, St. Paul, MN). Bioluminescent readings were acquired every 15 s and time to peak values (*T*_*max*_; min) were determined when the light intensity reached the maximum value of the curve (Fig. 1).

### Conventional LAMP

For comparison, a conventional *invA*-based *Salmonella* LAMP assay was run as described previously [14] using the Sal4 primer set. The reaction was conducted at 65 °C for 60 min and terminated at 80 °C for 5 min in a real-time turbidimeter LA-500 (Eiken Chemical Co., Ltd, Tokyo, Japan). Turbidity readings at 650 nm were obtained every 6 s and time threshold values (*T*_*t*_; min) were determined when the turbidity increase measurements exceeded a threshold value of 0.15.

### 3M MDA *Salmonella*

The 3M MDA *Salmonella* assay was performed following the manufacturer’s instructions. Briefly, 2 μl of DNA template and 18 μl of molecular-grade water (in pure-culture testing) or 20 μl of spiked food/feed homogenate or enrichment broth (in food/feed testing) were added into a lysis tube, heated at 100 °C for 15 min, then cooled for 10 min in a pre-chilled chill block. After mixing and holding at room temperature for 5 min, 20 μl of the lysates was transferred to a reagent tube and a matrix control tube (in food/feed testing only) containing lyophilized reagents. The reaction was carried out at 60 °C for 75 min in the 3M MDS100 instrument. *T*_*max*_ values were determined similarly as in the LAMP-BART assay.

### Specificity and sensitivity

For specificity testing, DNA templates of the 151 *Salmonella* and 27 non-*Salmonella* strains were prepared by heating at 95 °C for 10 min. Aliquots (2 μl) were subjected to the three assays (LAMP-BART, conventional LAMP, and 3M MDA *Salmonella*) and repeated twice.

Assay sensitivity (limit of detection) was determined by using 10-fold serial dilutions of *S*. Typhimurium LT2 cultures. DNA templates were prepared from stationary-phase cultures as described previously [39]. Aliquots were tested by all three assays and repeated five times. Detection limits were defined as the lowest concentrations that tested positive in all five repeats.

### Assay evaluation in spiked food and feed samples

Five types of food (cantaloupe, ground beef, ground turkey, shell eggs, and tomato) and three types of feed (cattle feed, chicken feed, and dry dog food) were examined. The food items were obtained from a local grocery store and processed as described previously [31, 32, 40]. The feed items were obtained from a local feed store and 25-g samples were apportioned for analysis. All food and feed samples were analyzed for the presence of *Salmonella* by conventional culture method [7] and confirmed negative samples were used for the following spiking experiments.

To determine assay sensitivity in each food or feed type, test portions (25 g) were inoculated with 1.5 ml of 10-fold serial dilutions of respective *Salmonella* overnight cultures (Table 3) as previously described [39], resulting in spiking levels between 10^8^ and 10^4^ CFU/25 g. Another sample was included as the uninoculated control, for which aerobic plate counts were performed. All samples were air-dried in a laminar flow biosafety cabinet for 2 h then homogenized with 225 ml of buffered peptone water (BPW; 3M Food Safety) for 2 min at high speed (260 rpm) in a food stomacher (Model 400; Seward Laboratory Systems, Inc., Davie, FL). For 3M MDA *Salmonella*, 20 μl of the homogenate was processed following the manufacturer’s instructions and the assay was repeated three times. For LAMP-BART and conventional LAMP, 1 ml of the homogenate were first centrifuged at 900 × *g* for 3 min to remove large particles, the supernatant transferred to a fresh tube, followed by another centrifugation at 16,000 × *g* for 3 min. The pellets were suspended in 100 μl of PrepMan Ultra sample preparation reagents (Applied Biosystems, Foster City, CA), heated at 95 °C for 10 min, cooled down to room temperature, and centrifuged again at 12,000 × *g* for 2 min. The supernatants (2 μl) were used for the assays, which were repeated three times.

The assay’s capability to detect low levels of *Salmonella* cells in these food and feed types were also evaluated. For this application, each test portion was inoculated similarly with respective *Salmonella* overnight cultures at 10^0^ to 10^1^ CFU/25 g. After homogenization in 225 ml of pre-warmed BPW, the samples were incubated at 35 °C for 24 h. Aliquots of the enrichment broth were processed similarly as described above and tested by all three assays. The low-level detection experiment was independently repeated three times.

### Data analysis

Means and standard deviations of *T*_*max*_ for LAMP-BART and 3M MDA *Salmonella* and *T*_*t*_ for conventional LAMP were calculated by Microsoft Excel (Seattle, WA). The values were compared using the analysis of variance followed by post-hoc multiple comparisons using the Least Significant Difference (LSD) test (v9.1; SAS for Windows, Cary, NC) and differences were considered significant when *P* < 0.05. Standard curves to quantify *Salmonella* in pure culture were generated by plotting *T*_*max*_ or *T*_*t*_ values against log CFU/reaction, and linear regression was calculated using Microsoft Excel. Quantification capabilities of the assays were derived based on the correlation coefficient (*R*^*2*^) values from the standard curves.

## Abbreviations

APS, adenosine 5′ phosphosulfate; BART, bioluminescent assay in real-time; dNTP, deoxynucleoside triphosphate; DTT, dithiothreitol; GMO, genetically modified organism; LAMP, loop-mediated isothermal amplification; LSD, least significant difference; MDA, molecular detection assay; MDS, molecular detection system; NAAT, nucleic acid amplification test; PVP, polyvinylpyrrolidone; qPCR, real-time quantitative PCR.
